# Physical Characterization of Sintered NiMnGa Ferromagnetic Shape Memory Alloy

**DOI:** 10.3390/ma13214806

**Published:** 2020-10-28

**Authors:** Francesca Villa, Adelaide Nespoli, Carlo Fanciulli, Francesca Passaretti, Elena Villa

**Affiliations:** Consiglio Nazionale delle Ricerche—Istituto di Chimica della Materia Condensata e di Tecnologie per l’Energia (CNR-ICMATE Sede di Lecco), via G. Previati 1/e, 23900 Lecco, Italy; adelaide.nespoli@cnr.it (A.N.); carlo.fanciulli@cnr.it (C.F.); francesca.passaretti@cnr.it (F.P.); elena.villa@cnr.it (E.V.)

**Keywords:** ferromagnetic shape memory alloys, sintering, martensitic transformation, thermal properties, internal friction, electrical properties

## Abstract

The present work focused on the microstructural, thermal, electrical, and damping characterization of NiMnGa samples produced through a powder pressing and a sintering process; the effect of sintering times and of the starting powder size were evaluated. Moreover, an observation of the evolution of martensitic transformation typical of NiMnGa ferromagnetic shape memory alloy was conducted in comparison with the cast material behavior and in correlation with the material densification. The optimum powder size and sintering time for the process, i.e., 50 µm or lower and 72 h, were identified considering the investigated physical properties of the sintered samples in comparison to the cast material. The corresponding sample showed the best compromise between density, thermal and electrical properties, and damping and functional behaviour. In general, the outcomes of this study could be the basis of a useful tool for production processes that include a sintering step as well as being a starting point for the evaluation of an alternative low cost fabrication method of this alloy.

## 1. Introduction

NiMnGa alloys are functional materials that perform a coupling between magnetic and structural order and this feature leads to a large magnetic-field-induced strain (MFIS), up to 10% in single crystals, caused by the rearrangement of the twin boundaries induced by an applied magnetic field [1]. In addition, materials with specific compositions could exhibit magnetic superelasticity or shape memory effect and magnetocaloric effect, when exposed to a magnetic field. Ternary Ni_50_Mn_25_Ga_25_ alloy and off-stoichiometric compounds have attracted increasing interest in several application fields including sensor and actuator development, due to their functional properties which are strictly correlated to crystal structure and phase transformation [1]. In recent years, the modulation of the grain structure through shape and size in NiMnGa alloys has attracted increasing interest in order to induce an improvement of their functional properties, in particular the magnetic field induction strain (MFIS). Furthermore, the presence of space between the grains and their particular shape is not a trivial condition for the development of smart behaviour as well as for for polycrystalline samples [2]. Hence, increasing attention has been devoted to different sorts of sintering routes. The first scientific focus for this development was the preparation of powders of the alloys and the study of the relation between the size, the structural properties, and the martensitic transformation [3]. Particularly, hindrance of transformation by means of residual strain induced by mechanical ball milling and its residual strain recovery by thermal treatment have been discussed [3]. In recent years, the introduction of several additive manufacturing processes has opened new possibilities for the preparation of sintered NiMnGa materials starting from powders. However, the high brittleness of NiMnGa alloys has not been completely solved by these kinds of processes [2] so the new perspectives introduced by innovative additive sintering methods have become of great interest. In fact, sintering processes assisted by the use of binders or inks have been largely investigated and developed in relation to these novel additive manufacturing techniques [4,5,6,7,8,9]. In these cases, the production of novel sizes and complex geometries, that could not be obtained by traditional processes, have been achieved. In addition to innovative shapes, these additive manufactures allow new microstructures that have caught the attention of most researchers. Particularly, scientific investigation, has been mainly focused on understanding the effect of these new microstructures on crystal grains, martensitic structure, and physical properties [8,10].

The thermoelastic martensitic transformation (TMT) could be considered a useful tool for investigating new microstructures since it significantly changes with temperature and involves transformation energy correlating with the grain characteristics. Moreover, in the literature, studies of process parameters and microstructure can be found although at present, neither the devices or the measurements of functional performances (like strain recovery vs. temperature) have been proposed. Only a few examples on sintering processes and on composite structures development have been studied [11,12] and they have generally provided an overview of the physical characteristics and the secondary phases of these new type of materials and structures in correlation with the sintering process conditions [10,13]. Despite diffusion mechanisms being crucial issues in sintering routes, there are only a few studies about investigation of the interdiffusion behaviour in NiMnGa alloys that could be useful for the study of the evolution of transport mechanisms during powder compaction [14].

Since the already discussed previous studies [2] had found out that the effect of grain morphology is a crucial point for the optimization of functional properties in the case of polycrystalline NiMnGa alloys, new kinds of structures could now be investigated in order to verify the possible size effects caused, for example, by the presence of pores. Therefore, the present work aimed to preliminarily investigate and optimize a simple and low-cost fabrication method which could be a valuable alternative to additive manufacturing processes for the production of porous structures. Indeed, the adopted sintering route is a cheap and reproducible processing method since it could be widely and easily implemented. The research presents novel results on a Ni_50_Mn_30_Ga_20_ alloy produced by sintering on the investigation and characterization of the physical properties of the samples, obtained by a quite traditional and low cost process consisting of a cold pressing of polycrystalline powders followed by different sintering routes. This study’s ambition was, first, to point out the effect of process conditions, then the possible perspectives of the powder-based NiMnGa alloy, and finally to overcome the challenges. Furthermore, it could delineate a path for an optimization of the processing route to provide an effective alternative to cast materials.

## 2. Materials and Methods

Cast NiMnGa samples, of Ni_50_Mn_30_Ga_20_ nominal composition, were prepared by 5 arc melting cycles of the pure elements (electrolytic Ni 99.97%, electrolytic Mn 99.5% and Ga 99.99%) in stoichiometric ratio, in a non-consumable electrode furnace (Leybold LK6/45) (Leybold, Cologne, Germany). As-cast ingot was ground to powder in a planetary ball mill (Fritsch Pulverisette 4) (Fritsch, Idar-Oberstein, Germany) and the powder size selected by means of sieves. Densified pellets were produced by die pressing alloy powders with different average size (lower than 50 μm or between 50 and 100 μm) at 0.75 GPa at room temperature and sintered by a thermal treatment at 925 °C for 24, 72, and 168 h in an Ar atmosphere, followed by slow cooling in the furnace. Sintered pellets had the following dimensions: approximately 3 mm in height and 13 mm of diameter. Table 1 provides a summary of the prepared sintered samples.

A polycrystalline solubilized sample obtained from ingot was thermal treated at 800 °C for 72 h and used as a useful comparator in the discussion of results. Time and temperatures of the thermal treatments for both the ingot and sintered samples were defined according to previous studies of our research group on NiMnGa alloys [15].

The microstructural observation of the sintered alloy was performed on polished samples by Leitz-ARISTOMET optical microscope (Leica Microsystems, Wetzlar, Germany) and Scanning Electron Microscope (SEM) LEO 1430 (Zeiss, Oberkochen, Germany) equipped with INCA Energy 200 dispersive X-Ray spectroscopy (EDS) (Oxford Instruments, Abingdon-on-Thames, UK) for the chemical composition investigation. Since the samples were characterized by high porosity and surface roughness, it was not possible to perform reliable density measurements via the Archimedes method, which has been adopted in some previous studies [10]. In fact, the presence of both open and closed porosity produces large instability in the results of repeated measurements. Therefore, density was estimated through image analysis conducted on the optical micrographs collected in the central cross sections of the sintered samples. Mean density was calculated over 6 measurements obtained from different sample areas.

Pure thermal analysis was conducted by Differential Scanning Calorimeter (DSC) Q100 (TA Instruments, New Castle, DE, USA). with cooling and heating rate equal to 10 °C/min on cast and sintered samples.

Both Dynamic Mechanical Thermal Analysis (DMTA) and strain recovery measurements were performed by means of DMA Q800 dynamometer (TA Instruments, New Castle, DE, USA). in three-point-bending configuration on cast and sintered samples of approximately 1 mm thickness and 10 mm length. DMTA analyses were conducted under temperature control (2 °C·min^−1^) at a strain of 0.02% and at 1 Hz frequency; strain recovery tests were performed at constant loads (between 0.5 and 30 MPa) and under a temperature change rate of 5 °C min^−1^.

X-Ray Diffraction (XRD) analyses were conducted by means of X-Ray Diffractometer Panalytical XPert PRO (Malvern Panalytical, Malvern, UK) with spinner probe at room temperature (25 °C) and the obtained patterns were refined using Materials Analysis Using Diffraction (MAUD) software. Williamson-Hall (W-H) analyses were also performed on the collected XRD patterns. A preliminary indication of the effect of the preparation process was assessed through the comparison between the sample obtained from ingot and the C sintered specimen.

Finally, electrical resistivity measurements were performed by means of the collinear-four-point probe method, adopting the suitable geometrical correction factors [16] on samples approximately 1 mm thick in a *T* range between 30 and 150 °C, while *c_p_* measurements were conducted with modulated DSC with Q100 TA Instruments between 50 and 140 °C with isothermal steps at temperature intervals ranging from 5 to 20 °C. It is a known fact that the thermal and electrical properties of metals are related, and in the case of porous materials, some theoretical approaches have been developed in order to correlate the transport properties (thermal and electrical conductivity) with the level of porosity. In [17] for example, a combination of general effective media (GEM) method and Weidemann–Franz relation (Equation (1)) was adopted in order to analyse thermal and electrical conductivities experimentally measured on porous Cu samples.

(1) $$\kappa_{eff}=L\times \sigma_{eff}\times T$$

where *κ_eff_* and *σ_eff_* are effective thermal and electrical conductivity respectively, *L* represents the Lorentz number and *T* is the absolute temperature. In the cited study, experimental results showed a general decrease of electrical and thermal conductivity by increasing the porosity and the trend was well fitted by theoretical predictions. In the present work, the thermal conductivity is derived from electrical resistivity data adopting Equation (1), while the specific heat is the experimentally measured property determined by modulated DSC. In the following discussion we analyse these results considering the theoretical correlation shown in Equation (2). In fact, the two thermal properties are related by means of thermal diffusivity which measures the ability of a material to conduct thermal energy in combination with its ability to store it:

(2) $$\alpha =d\times \frac{c_{p}}{\kappa_{eff}}$$

where *d* is the bulk density of the sample.

## 3. Results and Discussion

### 3.1. Morphological Investigation

**Figure 1:**
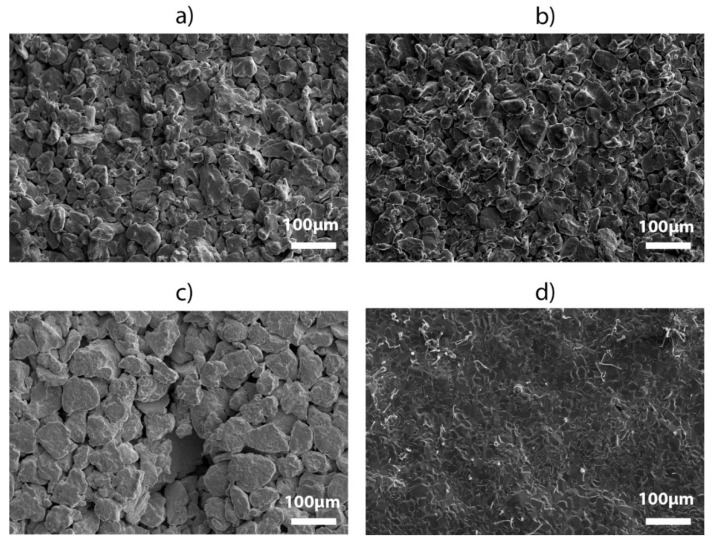
SEM micrographs registered for (**a**) sample A, (**b**) sample B, (**c**) sample C, (**d**) sample D.

**Figure 2:**
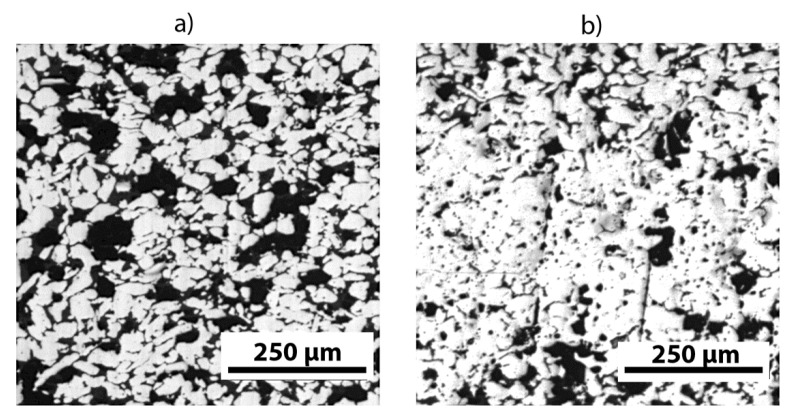
Optical micrographs registered for (**a**) sample A and (**b**) sample B. Sintered pellet dimensions: 3 mm (height) and 13 mm (diameter).

**Figure 3:**
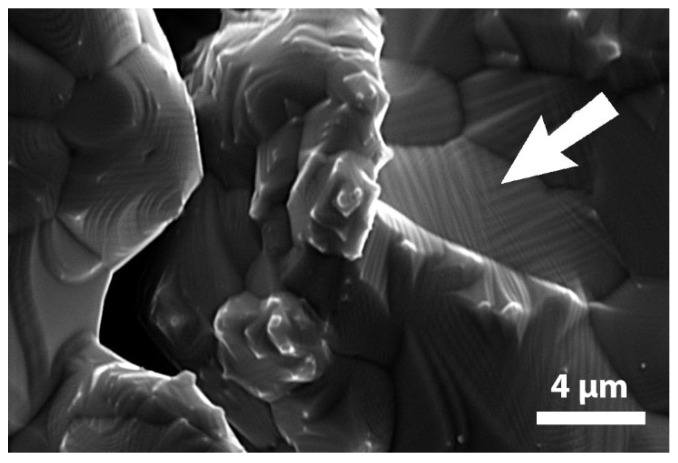
High magnification SEM micrograph of sample B, registered at room temperature. The arrow indicates martensitic twins.

A morphological analysis was carried out through microscope observations on sintered samples in order to evaluate the densification achieved by the different sintering treatments and the microstructure evolution after the annealing processes. The results of optical and scanning electron microscopy are reported in Figure 1, Figure 2 and Figure 3.

Figure 1 depicts the SEM micrographs of the four sintering samples at the same magnification. Comparing the four morphologies, the first result on the effects of the sintering time on material densification could be carried out. It was found that the material densification grows with the sintering time reaching the highest level with sample D, that is after 168 h treatment (Figure 1d). However, EDS compositional analyses on sample D detected a drastic decrease of Ni and Ga content and a severe degradation of the ternary NiMnGa system meaning that too long a sintering time leads to a lack of composition due to extreme diffusion and loss of these elements. In addition, also oxidation phenomenon was detected by EDS analysis probably caused by the insufficient atmosphere protection during thermal treatment over such long times. In fact, the measured average chemical composition, without considering the oxygen content, was Ni_18.1_Mn_80.7_Ga_1.2_ indicating a loss of 32 at% Ni and 18 at% Ga. As a consequence, this sample was not considered for further characterizations. Comparing samples A and B, coming from the same powder size (≤50 μm) but respectively processed for 24 and 72 h (Figure 1a,b), it is possible to observe that an increase in time of the sintering process leads to a more compact microstructure, with particles that are fused together at several points causing a large extent of necking phenomena (sample B). On the other hand, in sample A, the particles show a lack of sintering due to the shorter processing time than that of sample B, not enabling effective necking and particles coalescence, resulting in interconnected pores and detachment. Finally, it is possible to notice the different microstructure of sample C (Figure 1c) with respect to other samples, probably coming from the lower initial densification of the starting powders due to both the narrower size distribution and the larger powder size. In this case, the grains are poorly sintered leaving a large extent of voids that prevents particles coalescence, hence necking.

A qualitative evaluation of the relative density, expressed in percentage of voids, was conducted to confirm the morphological investigation: as expected, the highest density level (19 ± 2.4% of voids) was obtained for the sample sintered from finer powders for 72 h (sample B), with a greater extent of compactness in the central part of the cross section as shown in Figure 2a. On the other hand, the sample with the lowest sintering time, i.e., 24 h, (Figure 2b) achieved the lowest relative density (29 ± 2.1% of voids).

Microstructural analysis was completed by the high magnified SEM observations registered at room temperature: the presence of martensitic structure at this temperature was confirmed since it was possible to notice the presence of several thin martensitic twins within grains as indicated by the arrow in Figure 3 for sample B.

### 3.2. XRD Analysis

**Figure 4:**
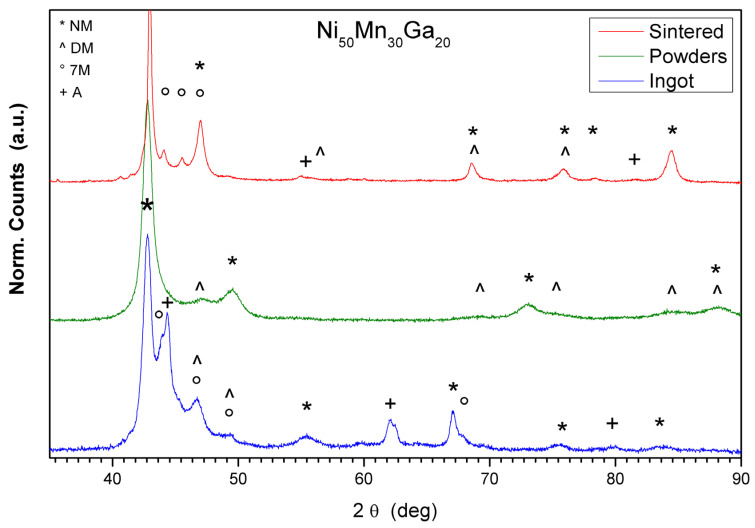
Normalized and translated XRD patterns collected at 25 °C from as cast ingot, milled powders, and sintered pellet (sample C). Different symbols are used to identify the peaks corresponding to the non-modulated martensite (NM), the distorted monoclinic structure (DM), the long range modulated martensite (7M) and the austenite (A) observed in the samples.

XRD analyses at the different steps of sample preparation were performed in order to study the effects of the processing on the microstructure of the material. XRD patterns collected from an as cast ingot, a milled powder sample, and a sintered pellet are reported in Figure 4: all the patterns display a pure Ni_50_Mn_30_Ga_20_ phase with no secondary phases or precipitates. In the figure, the peaks corresponding to the different crystal phases are marked with different symbols. The *, +, ° and ^ symbols are used for non-modulated martensite, austenite, distorted monoclinic, and modulated martensite phases respectively. The difference visible in the peak positions for the same phase can be associated with the structural distortions induced in the material by the applied processing. In order to investigate in depth the difference in the three patterns, Rietveld refinement and Williamson–Hall analyses were performed.

The refinement of the XRD pattern collected on a cross section of the as cast ingot, reveals the presence of a low quantity of austenite (4%) and a primary martensitic phase (96%). The pattern shows the presence of the non-modulated martensitic structure with a short range order and a superstructure proper of the seven-layered modulated (7M) martensite. The cell parameters for both the structures used for the refinement correspond to the data reported in the literature [18,19] for non-modulated (NM) and 7M structures. The observed long order is coherent with the results obtained by Williamson-Hall analyses performed on the refined pattern. The results show a crystallite size in the order of 68 nm with practically null structural strain. Despite martensite being the stable structure for this alloy at 25 °C, the relatively low quantity of austenitic phase revealed through XRD refinement was probably preserved during the quench process applied to the ingot.

Milled powders were investigated to evaluate the effects of the milling process on the material. The first evidence was an enlargement of the main peaks and the absence of some peaks observed in the as cast ingot pattern, such as the ones observed in the range 55–70°. This is in agreement with the fact that data are refined by martensite structures only. Here, the NM phase with tetragonal structure appears distorted mainly along the c axis (*a* = 3.702 Å, *c* = 7.259 Å). A quantitative evaluation of the phases found that NM martensite accounts for 36% of the sample. The main martensitic structure has a monoclinic structure and represents 64% of the sample. The monoclinic cell results in being close to the values reported in the literature in terms of lattice parameters, but a relevant increase of the tilted angle from 90.3° up to 101.5° is observed. Despite the hard processing applied to the material through the high energy ball milling, the W–H plot calculated on the XRD pattern, suggests a low structural strain (2%) associated with the powders. This is probably due to the tendency to cleavage of NiMnGa alloy, leading to an effective powder size reduction without internal strain storage. Moreover, a rearrangement of the martensitic structures into the powder grains could be another mechanism supporting a limited storage of internal mechanical energy. The resulting size of the crystallites, calculated by the Sherrer formula based on W–H plot results, is in the order of 8.5 nm, largely reduced with respect to the starting as cast ingot.

The same analyses were performed on the pellet 72 h sintered at 925 °C (sample C). The material shows again a low content of residual austenite (refined pattern gives a content value in the order of 1%): this value is coherent with the slow cooling route followed by the sample which allowed the complete structural transition to the martensitic form. The martensitic phase shows the presence mainly of short range order with a net predominance of tetragonal structure (close to 62% of the phase). At the same time, the pattern reveals the presence of monoclinic structure: in this case the structure is more regular and less tilted with respect to the one observed in the milled powders so that the tetragonal structure does not display a significant modification of the lattice parameters (*a* = 3.872 Å, *c* = 6.601 Å). Weaker evidence with respect to the cast material of the long range modulated martensitic structure appears in the pattern and the refinement suggests the presence of 7M modulated martensite, as reported in the inset in Figure 4. The good agreement of the lattice structure to the one reported for this phase suggests the complete recovery of the material induced by the thermal treatment after the ball milling process. Further data supporting this statement comes from the Williamson-Hall analysis showing a limited growth of the crystallites reaching 14.5 nm and a residual strain close to zero. The limited growth of the crystals size, despite the high temperature achieved during the sintering process, is probably due to the absence of pressure applied to the pellet during the treatment. The scarce intimal contact achieved by the grains during the process limited the grain growth. This leads to a low density sintered pellet in which the structure is fully recovered. Despite the results of Williamson-Hall analyses showing the absence of internal stresses in the structure, the crystallite domains display a limited growth with respect to the average grain size. Such evidence suggests the presence of domains boundaries, probably dislocations or line defects along the crystal structure not released during heat treatment, working as barriers to coherent merging of the consecutive domains.

### 3.3. Thermal and Mechanical Characterization

**Figure 5:**
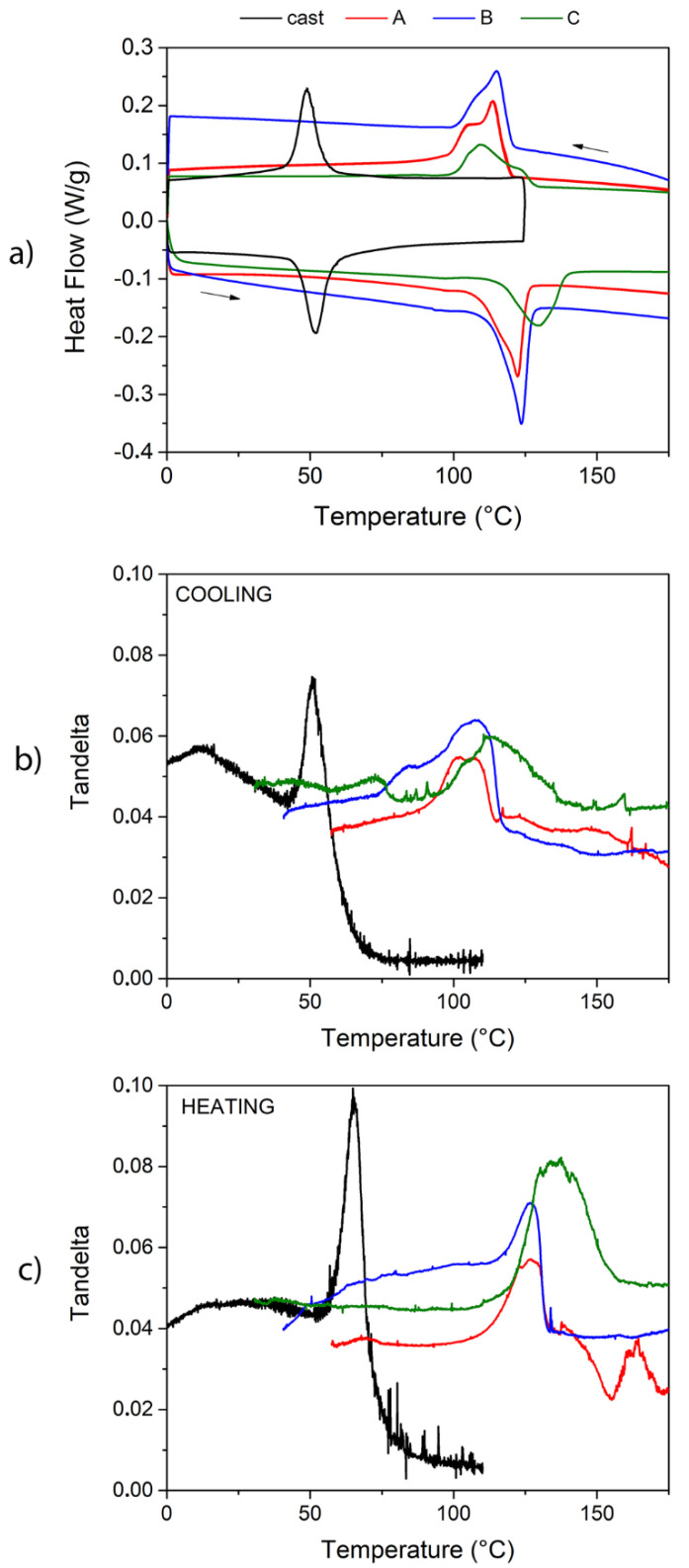
DSC analysis results (**a**) and DMTA curves vs. temperature in heating (**b**) and cooling stage (**c**) for the investigated samples.

In order to investigate the influence of grain size and compactness of the sintered samples on the thermally activated functional properties, pure thermal and thermo-mechanical measurements were conducted in comparison with cast samples. The results of DSC tests are shown in Figure 5a: the transformation temperatures and enthalpies extracted from the curves are reported in Table 2.

The results display the shift of martensitic transformation towards higher temperatures in the case of sintered samples: this could be first ascribed to the different treatment temperature and to the higher energy barriers provided by the defects generated during the processing in the intergranular regions. Indeed, during the ball-milling the heat released leads to an incoherent growth of crystallites that cannot be significantly recovered during the subsequent sintering process, leaving several defects. As obtained by XRD analyses, the domains of structural coherence, i.e., the crystallites, are small and do not show a long-range periodicity, proper for this system, because of the energy barriers to regular merging during the sintering process. As observed from the peak secondary structures of martensite in XRD analyses, only weak evidence of martensite long range periodicity is achieved in the sintered materials with respect to the one measured for the cast sample. In the sintered case, the granular structure observed in microstructure analyses does not coincide with the crystalline domains being smaller. Hence, the material turns out to be fragmented and not continuous. The presence of these energy barriers in the structure of sintered samples was not detected by XRD analysis being strictly related to the crystal lattice; on the other hand, some evidence of this phenomenon could be observed in the thermal and mechanical analyses, reported in Figure 5, that show the nature of the TMT which is deeply related to the grain structure. The DSC peaks related to sintered samples are not only shifted in temperature, but quite broader than the cast ones due to morphological disorder and to the high amount of voids present in the sintered material. The double shape of the peaks of sintered samples, and the shift in temperature with respect to the cast material could reflect the dispersion in temperature of the martensitic transition, in the case of NiMnGa alloy maybe due to different energy domains of the martensitic structure related to different levels of energy barriers. Furthermore, for the samples sintered from finer particles (samples A and B) the peak position is approximately the same, while for the one with coarser particles (sample C) the peaks are broader and shifted towards slightly higher temperatures, perhaps due to the coarser granulometry that could enhance the effect of inter and intragranular energy barriers, voids, and defects, since the chemical composition checked by EDS is unaltered.

After the pure thermal characterization, a preliminary mechanical investigation of functional properties activated by a thermal change was provided by different tests: Dynamic Mechanical Thermal Analysis (DMTA) and strain recovery measurements.

The Tandelta parameter obtained from DMTA measurements is a precise probe of the microstructural condition of the material [20]. Observing DMTA results reported in Figure 5b,c, it is possible to notice that the cast material gives a good damping performance during the martensitic and reverse transition with peak values that reach 0.075 (cooling stage) and 0.1 (heating stage), but also in the martensitic state the value of Tandelta is noticeable with values between 0.05 and 0.06 for a wide range of temperatures below the martensite finish temperature (M_f_). On the other hand, the peak values obtained for sintered materials are generally lower and shifted towards higher temperatures, as confirmed by DSC analysis: Tandelta peaks range between 0.055 and 0.065 for the cooling stage and between 0.055 and 0.07 in the heating stage. The martensitic values of IF are comparable to the cast sample for the sample B and C and slightly lower for sample A. Furthermore, the sintered samples show high average values of Tandelta (between 0.03 and 0.05) also in parent austenitic phase, while for the cast material this value drops below 0.01 as is usually observed for ordered austenitic structures in shape memory alloys. Indeed, the particular microstructural condition of sintered samples allows the combination of two different contributions to the damping performance out of the transition region: the damping due to the martensitic twin structure and the mechanical energy dissipation effect of the particular sintered grain structure characterized by coexistence of joined grains, necks, voids, and defects. This is observed particularly for sample C which presents the largest extent of voids (see Figure 1).

**Figure 6:**
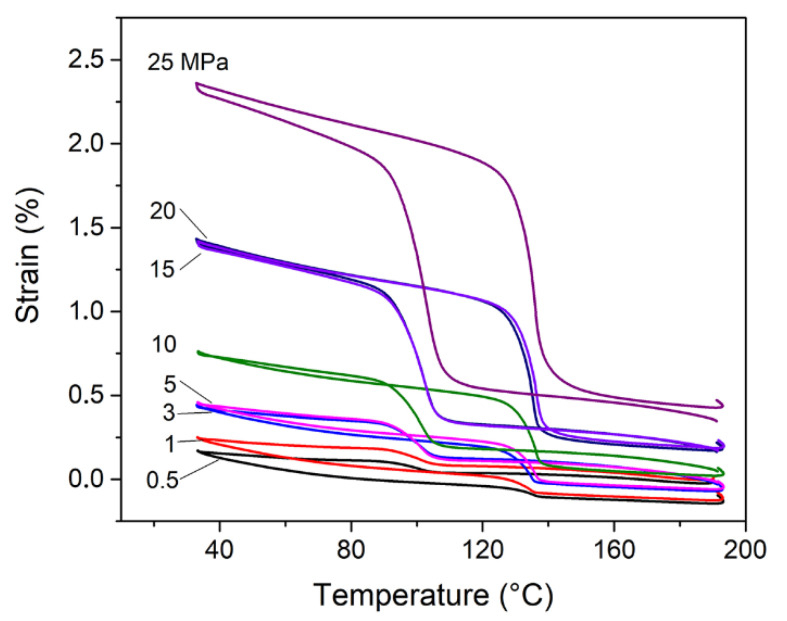
Strain recovery measurements under constant loads vs. Temperature of the sample B. Curves are labelled with constant loads values in MPa.

Among sintered samples, the best strain recovery properties under constant loads are shown by the sample B since higher density is the crucial issue in the determination of mechanical behaviour of the sintered materials. In Figure 6, we report the behaviour of sample B which can withstand a maximum stress of 25 MPa and on a thermal cycle it is able to recover approximately 2% of strain without residual strain. It is also interesting to note a preliminary inverse strain recovery behaviour at low loads, i.e., for the curves registered under 0.5 and 1 MPa, due to microstructural energy barriers introduced in the sintered material [21].

### 3.4. Electrical and Thermal Properties

**Figure 7:**
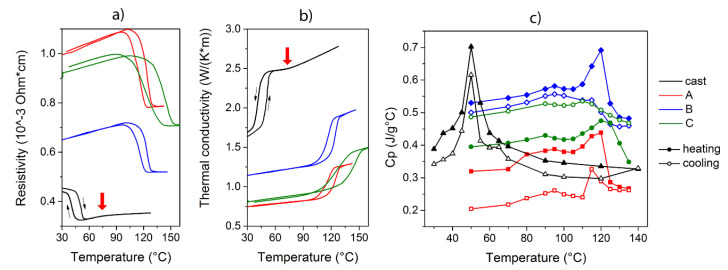
Resistivity vs. Temperature measurement (**a**), calculated thermal conductivity vs. Temperature (**b**) and specific heat vs. Temperature measurement (**c**) for the investigated samples. The red arrows in (**a**,**b**) indicate the change in slope observed for cast sample.

Finally, a thermal and electrical characterization of the sintered materials was carried out by means of electrical resistivity (*ρ*) and specific heat (*c_p_*) measurements, reported in Figure 7a,c respectively. An indication of the thermal conductivity (Figure 7b) was derived from Equation (1), since ρ is the inverse of the electrical conductivity. In this way, it was possible to delineate a correlation between electrical and thermal properties of the sintered samples.

Electrical resistivity measurements give evidence of the transition occurring corresponding to the martensitic transformation. It is possible to observe an initial increasing trend of the resistivity by increasing the temperature as typically observed in metals, while when we approach the transformation temperature the resistivity decreases until the material is completely transformed. The temperature hysteresis between direct and inverse transformation (upon heating and cooling) is clearly visible for both sintered and cast material. For the cast material, the change in the slope of the increasing trend of the resistivity and thermal conductivity detected before the TMT, around 75 °C, could be evidence of the Curie transition. The comparison of the resistivity values is performed not to obtain the precise value of intrinsic resistivity of the material, but for a check of the curves’ trends and their relative comparison that could be evidence of different microstructures. As expected, the geometrical error due to the particular microstructure with voids, typical of sintered samples, influences the absolute value of this physical parameter. This characterization allows having a picture of the effect of these microstructures on the transmission of heat and electrical current. In this way, only the comparison of the curves of different samples is useful and not the values themselves. In this case, the resistivity, the heat capacity and the heat conductivity result as a feedback of the effect of process parameters on the preparation of sintered samples and as a first indication of the functional properties expected starting from different preparation routes. As expected, the resistivity of the cast sample is the lowest and it increases on increasing the porosity in sintered samples, due to the growing amount of voids and inhomogeneity acting as obstacles to the current flow.

On the other hand, as expected from Equations (1) and (2), specific heat measurements illustrate an opposite trend: sintered samples characterized by higher porosity present lower specific heat. It is possible that the effect of pores is an obstacle to electrical transmittance but at the same time, favours the storage of heat rather than its transmission through the material, resulting in a decrease of the specific heat. Furthermore, it is possible that the presence of small and short-range ordered crystal structures, previously discussed, could influence, at a macroscopic scale, the heat capacity of the system. A discrepancy is noticeable between *c_p_* values in cooling and heating stages, particularly for samples A and C: it is an experimental effect due to the disordered morphology and voids, filled with air, characterized by poor thermal conductivity, that leads to different heating and cooling routes in sintered samples. Considering the cast material, it is possible to observe a well-defined peak corresponding to the martensitic transition with a quite small hysteresis between the heating and cooling stage, as confirmed by the DSC curves. A slight step is detected around 65 °C in the cooling stage of cast alloy and this could be thermal evidence of the Curie transition, that may be displayed at lower temperatures with respect to the electrical measurements.

Further analysis of the conducted measurements allowed an interesting comparison among the hysteresis due to TMT, visible in resistivity and thermal analysis measurements. It could be a useful comparison of the description of this energy dissipation from two physical parameter points of view.

**Figure 8:**
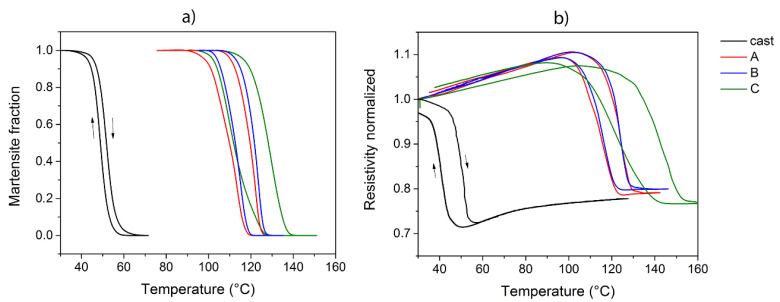
(**a**) Hysteresis cycles from DSC and (**b**) hysteresis cycles obtained from resistivity measurements.

The hysteresis cycles reported in Figure 8a were evaluated by integration of the DSC peaks related to the thermoelastic transformation. In this way, it was possible to obtain the evolution of the martensite volume fraction during the transformation. The thermal hysteresis derived from DSC was compared to the hysteresis measured during the resistivity measurements normalized with respect to the resistivity value at 30 °C reported in Figure 8b.

The hysteresis of the sintered samples is approximately the same for thermal and electrical measurements, while it is possible to observe a difference in the case of the cast material. Indeed, the electrical hysteresis of cast alloy is more pronounced than the thermal one, since the nature of the two measurements is different and the physical parameters seem to act differently on the cast material. A more evident difference between the heating and cooling route is shown from the electrical point of view and also possible evidence of the Curie transformation is shown by these kinds of measurements.

## 4. Conclusions

In the present work, NiMnGa samples were prepared through pressing and sintering processes starting from milled powder with different granulometry. The effects of the sintering time were assessed through physical and mechanical analyses. The studied process was demonstrated to be a valid simple and low cost route to obtain samples with interesting functional properties that could be suitable for optimization and for the development of applications for small scales devices. Indeed, microscopy analyses allowed the investigation of the morphology and the microstructure of the samples and the results were correlated to thermal, damping, and electrical outcomes. All the presented analyses allowed delineation of a fairly complete characterization of the thermoelastic martensitic transformation of sintered NiMnGa, hence the activation of functional properties, from several points of view. It was found that microstructure plays a major role on material performance, with respect to thermal hysteresis or electrical conductivity; it seems that the electron mobility is significantly affected by the martensitic and austenitic structure. Conversely, the nucleation and the shrinking of martensite are efficiently revealed during the heat release and absorption measures for the cast material and the TMT shows a very narrow hysteresis in thermal characterization. On the other hand, electrical measurements may give more evidence of the Curie transition. Furthermore, the X-ray diffraction allowed a complete description of the martensitic structure and the residual strain after the milling process. In detail, we may conclude that the sample sintered from finer particles (size < 50 μm) for 72 h at 925 °C (sample B) presented the best compromise between achieved density, thermal and electrical properties, and damping and functional behaviour with respect to cast material. The present study investigated a simple and low-cost process for the sintering of NiMnGa alloy and could represent a useful guideline for future studies on these materials and their functional properties. Indeed, the optimization of the technique and the characterization of the materials were conducted exhaustively and it was possible to correlate the process parameters with the physical properties of the achieved structures. Moreover, part of our future work will be focused on further research and experimental analysis on the activation, optimization and characterization of the functional properties of NiMnGa porous alloys.

## Figures and Tables

**Table 1:** Description of sintered samples.

Sample	Size of Grain Powders	Sintering Time at 925 °C
A	≤50 μm	24 h
B	≤50 μm	72 h
C	50 μm < size ≤ 100 μm	72 h
D	≤50 μm	168 h

**Table 2:** Results of DSC analysis of the investigated samples.

Sample	A_s_ (°C)	A_f_ (°C)	ΔH_HEAT_ (J g^−1^)	M_s_ (°C)	M_f_ (°C)	ΔH_COOL_ (J g^−1^)
cast	45.4	57.1	6.4	55.7	42.9	−6.9
A	108.4	125.6	8.2	119.7	98.2	−8.0
B	113	127.1	9.5	120.1	101.2	−8.9
C	115.4	137.7	7.7	128.7	101.1	−6.0

## Data Availability

The raw/processed data required to reproduce these findings cannot be shared at this time as the data also forms part of an ongoing study.
